# Differences in hepatocellular carcinoma risk, predictors and trends over time according to etiology of cirrhosis

**DOI:** 10.1371/journal.pone.0204412

**Published:** 2018-09-27

**Authors:** George N. Ioannou, Pamela Green, Elliott Lowy, Elijah J. Mun, Kristin Berry

**Affiliations:** 1 Division of Gastroenterology, Veterans Affairs Puget Sound Healthcare System and University of Washington, Seattle, Washington, United States of America; 2 Department of Medicine, Veterans Affairs Puget Sound Healthcare System and University of Washington, Seattle, Washington, United States of America; 3 Health Services Research and Development, Veterans Affairs Puget Sound Healthcare System, Seattle, Washington, United States of America; 4 Department of Internal Medicine, University of Washington, Seattle, Washington, United States of America; Drexel University College of Medicine, UNITED STATES

## Abstract

**Background and aims:**

Hepatocellular carcinoma (HCC) risk is high in cirrhosis. We sought to describe differences in HCC risk, predictors and trends over time according to etiology of cirrhosis.

**Methods:**

We identified 116,404 patients with cirrhosis diagnosed between 2001–2014 in the VA healthcare system and determined incident HCC cases occurring from the date of cirrhosis diagnosis until 01/31/2017. Patients were divided by cirrhosis etiology into hepatitis C virus (HCV, n = 52,671), alcoholic liver disease (ALD, n = 35,730), nonalcoholic fatty liver disease (NAFLD, n = 17,354), or OTHER (n = 10,649).

**Results:**

During a mean follow-up of 4.3 years, 10,042 new HCC cases were diagnosed. Patients with HCV had >3 times higher incidence of HCC (3.3 per 100 patient-years) than patients with ALD (0.86/100 patient-years), NAFLD (0.90/100 patient-years) or OTHER (1.0/100 patient-years), an association that persisted after adjusting for baseline characteristics. HCC incidence was 1.6 times higher in patients with cirrhosis diagnosed in 2008–2014 (2.47/100 patient-years) than in 2001–2007 (1.55/100 patient-years).

Independent predictors of HCC among all cirrhosis etiologies included: age, male sex, Hispanic ethnicity, high serum alpha fetoprotein, alkaline phosphatase and AST/√ALT ratio and low serum albumin and platelet count. Diabetes was associated with HCC in ALD-cirrhosis and NAFLD-cirrhosis, and BMI in ALD-cirrhosis.

**Conclusions:**

HCC risk is 3 times greater in cirrhotic patients with HCV than ALD or NAFLD. HCC risk continues to increase over time in analyses extending to 2017 in cirrhosis of all etiologies. Multiple readily available risk factors for HCC were identified that were influenced by cirrhosis etiology and could be used to develop HCC risk estimation models.

## Introduction

Patients with cirrhosis have significantly increased risk of hepatocellular carcinoma (HCC) ranging from 1 to 8% per year[1]. The three most common etiologies of cirrhosis in the United States, which also account for the majority of HCC cases, are hepatitis C virus (HCV), alcoholic liver disease (ALD) and nonalcoholic fatty liver disease (NAFLD).

The risk of HCC appears to be higher in patients with HCV-related cirrhosis than cirrhosis related to ALD, NAFLD or other etiologies[2–4]. It is unclear whether this difference persists after adjustment for baseline characteristics that are risk factors for HCC. Furthermore, some HCC risk factors may be different in magnitude depending on the underlying liver disease responsible of cirrhosis, such as HCC risk factors that are particularly linked to a specific liver disease (e.g. obesity and diabetes, which are strongly associated with NAFLD) or risk factors that are only relevant for a specific liver disease (e.g. HCV genotype). Describing the most important HCC risk factors by cirrhosis etiology is important for the future development of HCC risk prediction models that can be used in clinical practice.

The distribution of cirrhosis etiologies is expected to change dramatically as HCV-cirrhosis begins to decline and NAFLD-cirrhosis continues to increase. Although HCC incidence appears to continue to increase over time[5], it is unclear whether HCC risk is still increasing among patients with cirrhosis caused by different underlying liver diseases.

We aimed to compare different liver diseases (HCV, ALD and NAFLD) with respect to HCC risk in cirrhotic patients and to determine the most important risk factors for HCC separately for patients with HCV, ALD or NAFLD-cirrhosis. We also aimed to compare trends over time in the incidence of HCC among patients with HCV, ALD or NAFLD-cirrhosis.

## Methods

### Data source and study population

The Veterans Health Administration is the largest integrated healthcare system in the United States, providing care at 168 VA Medical Centers and 1053 outpatient clinics and serving more than 8.9 million Veterans each year as of 2016[6]. We identified all patients whose cirrhosis was diagnosed for the first time during the 14-year period from 01/01/2001 to 12/31/2014 in the VA national healthcare system (n = 136,050) and retrospectively followed these patients until 01/31/2017 for incident HCC. Patients with a history of HCC before or within 90 days of the diagnosis of cirrhosis (n = 6531) and patients with a diagnosis of cirrhosis only after liver transplantation (n = 934) were excluded. Also, patients who died or underwent liver transplantation within 90 days of the diagnosis of cirrhosis or who had fewer than 90 days of follow-up (n = 12,181) were excluded leaving 116,404 patients in the current analysis. Data extended backward to October 1999 in order to allow determination of baseline characteristics and comorbidities and forward to 01/31/2017 so that all patients would have at least 25 months of potential follow-up for the development of HCC.

The VA uses a single, nationwide, comprehensive electronic healthcare information network (known as the Veterans Information Systems and Technology Architecture or VistA), which consists of nearly 180 applications of clinical, financial, administrative and infrastructure needs integrated into a single, common database. We derived electronic data from the VA Corporate Data Warehouse, a national, continually updated repository of data from VistA developed specifically to facilitate research[7]. We extracted patient demographics, inpatient and outpatient visits, problem lists, procedures, vital signs, diagnostic tests, laboratory tests, and pharmacy prescriptions.

The study was approved by the Institutional Review Board of the Veterans Affairs Puget Sound Healthcare System and the requirement to obtain informed consent was waived.

### Definition of cirrhosis

The diagnosis of cirrhosis was based on the presence of the ICD-9 codes for cirrhosis or complications of cirrhosis (gastroesophageal varices, encephalopathy, nonmalignant ascites) listed in **S1 Table**, recorded at least twice in any inpatient or outpatient encounter. This approach has been validated and widely used in VA-based studies by us[2, 5, 8–14] and others[15–17]. The diagnosis of cirrhosis using *a single* ICD-9 code in VA data has been shown to have a 90% positive predictive value (probability that cirrhosis is present among those with a code) compared to chart extraction[18]. By requiring the relevant ICD-9 codes to be recorded at least twice we have found that the positive predictive value increased to 97% in a random sample of 250 patients included in the current study.

### Cause of liver disease

Among patients with cirrhosis, we defined the following four etiologies of cirrhosis based on our previously published studies[5]:

1. HCV: Patients with a positive serum HCV RNA were categorized as HCV regardless of any additional etiologies
2. ALD: Patients with ICD-9 codes for alcohol use disorders (**S1 Table**) in the absence of serological markers of chronic HCV or HBV infection and in the absence if ICD9 codes for hemochromatosis, primary biliary cirrhosis, primary sclerosing cholangitis, and autoimmune hepatitis (**S1 Table**).
3. NAFLD: was defined in patients with diabetes (ICD-9 code 250–250.92, recorded at least twice[19]) or body mass index (BMI) ≥30 kg/m^2^ prior to the diagnosis of cirrhosis, who *did not have* HCV, ALD (defined as above) or ICD9 codes for hemochromatosis, primary biliary cirrhosis, primary sclerosing cholangitis, and autoimmune hepatitis. NAFLD-related cirrhosis does not have pathognomonic serological, radiological, or histological features—even hepatic steatosis is frequently absent after cirrhosis develops. Hence we adapted a clinical definition of NAFLD based on previous work[5, 20] that reflects the diagnostic process used in clinical practice, in which NAFLD is suspected in the presence of risk factors such as obesity and diabetes after exclusion of other etiologies.
4. OTHER: All other patients not meeting criteria above for HCV, ALD or NAFLD.

These definitions were mutually exclusive by design in order to allow comparisons by cirrhosis etiology. Since patients categorized under HCV may also have concomitant ALD, we performed secondary analyses subdividing HCV into those with and without concomitant ALD.

### Baseline patient characteristics

We extracted age, sex, race/ethnicity and all the baseline laboratory tests shown in **Table 1**, ascertained within 90 days of the diagnosis of cirrhosis. Body mass index (BMI) was calculated as the measured weight in kilograms divided by the square of the measured height in meters recorded within 6 months prior to cirrhosis diagnosis. We also determined the presence of signs of decompensated cirrhosis (defined as the presence of ascites, spontaneous bacterial peritonitis, encephalopathy, gastroesophageal varices or hepatorenal syndrome), type 2 diabetes mellitus, alcohol use disorders, substance use disorders and HIV infection based on appropriate ICD-9 codes recorded at least twice *prior to* the date of cirrhosis diagnosis in any inpatient or outpatient encounter (**S1 Table**). These ICD9-based definitions of cirrhosis, decompensated cirrhosis and other comorbidities have been widely used and validated in studies using VA medical records[5, 14–17, 19, 21].

**Table 1:** Baseline characteristics at the time of diagnosis of cirrhosis for all patients diagnosed with cirrhosis in the VA healthcare system from 2001–2014 (n = 116,404), presented according to cirrhosis etiology.

	ETIOLOGY OF CIRRHOSIS:				
	HCV N = 52,671	ALD N = 35,730	NAFLD N = 17,354	OTHER N = 10,649	P-value
**Age, yrs (mean**±**SD)**	57±7	60.3±9.22	66.3±9.9	65.6±12.3	<0.001
**Male (%)**	98%	98%	96%	96%	<0.001
**Race/Ethnicity (%)**					<0.001
White, non-Hispanic	58%	68%	72%	64%	
Black, non-Hispanic	23%	10%	7%	12%	
Hispanic	9%	8%	7%	5%	
Other	2%	2%	2%	2%	
Declined to answer/missing	9%	13%	13%	16%	
**BMI, Kg/m**^**2**^ **(mean**±**SD)**	28.5±5.54	28.1±5.92	32.7±6.29	26.5±4.7	<0.001
**Diabetes (%)**	29%	28%	74%	14%	<0.001
**Alcohol Use Disorder (%)**	51%	100%	0%	14%	<0.001
**Substance Use Disorder (%)**	34%	14%	1%	5%	<0.001
**HIV co-infection (%)**	3%	0.3%	1%	2%	<0.001
**HBV co-infection (%)**	1%	0%	0%	12%	<0.001
**Decompensated Cirrhosis (%)**	24%	34%	38%	39%	<0.001
Ascites (%)	10%	19%	21%	22%	<0.001
Encephalopathy (%)	2%	3%	2%	2%	0.6
Gastroesophageal Varices (with bleeding) (%)	2%	2%	2%	2%	0.07
Gastroesophageal Varices (without bleeding) (%)	7%	7%	10%	10%	<0.001
**HCV Genotype (%)**					N/A
Missing	52%	N/A	N/A	N/A	
Genotype 1	39%	N/A	N/A	N/A	
Genotype 2	4%	N/A	N/A	N/A	
Genotype 3	4%	N/A	N/A	N/A	
Genotype 4	0.4%	N/A	N/A	N/A	
**Laboratory Results, (mean**±**SD)**					
Alpha Fetoprotein (ng/mL)	60.2 ±223	23.7±109	31±101	21.2±-50	<0.001
Hemoglobin (g/dL)	13.4±2.24	12.6±2.32	12.6±2.21	12.8±2.25	<0.001
Platelet Count (k/μL)	136±77	175±101	163±92	181±107	<0.001
Creatinine (mg/dL)	1.04±0.84	0.99±0.59	1.31±1	1.2±0.92	<0.001
Bilirubin (g/dL)	1.5±1.8	2.4±3.5	1.2±1.3	1.6±2.5	<0.001
INR	1.26±0.34	1.37±0.49	1.38±0.61	1.35±0.54	<0.001
Albumin (g/dL)	3.3±0.7	3.2±0.8	3.5±0.7	3.4±0.8	<0.001
Alkaline Phosphatase (U/L)	119±69	146±102	124±96.1	152±147	<0.001
GGT (U/L)	206±291	328±453	155±204	221±323	<0.001
AST/√ALT ratio	10.6±5.8	10.6±6.6	7.0±4.9	8.2±5.0	<0.001

### Diagnosis of hepatocellular carcinoma

The diagnosis of HCC was based on the presence of ICD-9 code 155.0 and ICD-10 code C22.0 (the VA switched to ICD-10 codes on 10/1/2015) recorded at least twice. The ICD-9 code-based definition of HCC using VA records has been shown to have a positive predictive value of 84–94% compared to chart extraction[17, 22, 23] and has been widely used by us[2, 5, 13, 24] and other investigators[25–27].

### Statistical analysis

We used multivariable Cox proportional hazards regression to compare cirrhotic patients with HCV versus those with ALD or NAFLD with respect to the risk of developing HCC after cirrhosis diagnosis, adjusting for baseline characteristics. We also used multivariable Cox proportional hazards regression to determine the association between baseline characteristics at the time of cirrhosis diagnosis and the risk of developing HCC, separately for each cirrhosis etiology (HCV, ALD or NAFLD). Finally, we used Cox proportional hazards regression to compare the risk of HCC in patients with cirrhosis diagnosed in 2001–2007 versus those diagnosed in 2008–2014, separately by cirrhosis etiology limiting maximum follow-up to 5 years.

Follow-up started 90 days after the diagnosis of cirrhosis (since cases of HCC diagnosed within 90 days were almost certainly present at the time of cirrhosis diagnosis) and continued until the development of HCC or the date of liver transplantation or the date of death or the date of last follow-up in the VA, whichever occurred first. Follow-up could potentially extend up to 01/31/2017 such that all patients had a minimum potential follow-up of 25 months. Patients who did not develop HCC were censored at the date of death, liver transplantation or last follow-up. Patients with HCV who were cured as a result of antiviral treatment were censored at the date they achieved sustained virologic response.

Mortality is high in patients with cirrhosis and death can be a competing risk for HCC, that is, patients who are more likely to develop HCC may also be more likely to die beforehand thus masking the development of HCC. Liver transplantation, although much less common than death, can also be a competing risk for HCC. For this reason, we additionally performed competing risks[28] proportional hazards analysis to model the risk of HCC (which yields “sub-hazard” ratios instead of hazard ratios) while simultaneously accounting for the competing risk of death or liver transplantation.

## Results

### Comparison of patients according to cirrhosis etiology

The most common etiology of cirrhosis was HCV (45%) followed by ALD (31%), NAFLD (15%) and other etiologies (9%). Among patients with HCV, 51% also had a history of alcohol use disorders. Patients with NAFLD were substantially older, more likely to be white and, by definition, more likely to have diabetes and high BMI than other etiologies (**Table 1**). Conversely, patients with HCV were the youngest and most likely to be non-white.

Clinical manifestations of decompensated cirrhosis (ascites, encephalopathy or varices) were most common in NAFLD and OTHER etiologies and least common in HCV at the time of cirrhosis diagnosis. However, mean platelet count was lowest and mean serum alpha fetoprotein (AFP) level and AST/√ALT ratio were highest in patients with HCV.

Compared to patients who did not develop HCC, those who developed HCC were slightly older, had lower platelet count, higher serum AFP and were more likely to be male, Hispanic or diabetic (among ALD and NAFLD groups)–**Table 2**.

**Table 2:** Baseline characteristics of patients with or without a diagnosis of HCC during follow-up, presented according to cirrhosis etiology*.

	ETIOLOGY OF CIRRHOSIS:					
	HCV		ALD		NAFLD	
	No HCC N = 45,066	HCC N = 7,605	No HCC N = 34,342	HCC N = 1,388	No HCC N = 16,746	HCC N = 608
**Age, yrs (mean**±**SD)**	57.0±7.1	57.1±6.3*	60.2±9.3	62.3±7.8	66.3±10.0	66.4±8.1*
**Male (%)**	97.6	98.8	98	99.6	95.9	98.4
**Race/Ethnicity (%)**						
White, non-Hispanic	57.9	57.6	67.6	67.2	71.5	71.7
Black, non-Hispanic	22.8	22.6	9.9	5.4	6.7	2.5
Hispanic	8.3	11	7.5	16.7	6.7	12.7
Other	1.8	1.9	2.1	2.2	2	3
Declined to answer/missing	9.1	6.9	12.9	8.4	13.1	10.2
**BMI, Kg/m**^**2**^ **(mean**±**SD)**	28.5±5.6	28.3±5.1	28.0±5.9	30.0±5.7	32.7±6.3	33.3±5.8
**Diabetes (%)**	29	27.5	27	41.8	74.2	83.2
**Alcohol Use Disorder (%)**	51.3	50.6*	100	100	0	0
**Substance Use Disorder (%)**	34.9	32.0	14.5	8.1	1.5	1.2*
**HIV co-infection (%)**	2.8	1.6	0.3	0.2*	0.5	0.5*
**HBV co-infection (%)**	1.2	1.4*	0.0	0.0*	0.0	0.0*
**Decompensated Cirrhosis (%)**	23.9	22.6	34.1	32.9*	38.2	28.5
Ascites (%)	10.8	6.8	19.7	14.6	21.2	9.2
Encephalopathy (%)	2.6	1.9	3.3	3.0*	2.3	1.0
Gastroesophageal Varices (with bleeding) (%)	1.8	2.0*	2.3	3.1*	2.1	3.0*
Gastroesophageal Varices (without bleeding) (%)	6.3	9.1	6.7	9.9	10.3	13.0
**HCV Genotype (%)**						
Missing	52.4	49.9	N\A	N\A	N\A	N\A
Genotype 1	38.9	40.2	N\A	N\A	N\A	N\A
Genotype 2	4.3	3.4	N\A	N\A	N\A	N\A
Genotype 3	3.9	6.2	N\A	N\A	N\A	N\A
Genotype 4	0.4	0.3	N\A	N\A	N\A	N\A
**Laboratory Results, (mean**±**SD)**						
Alpha Fetoprotein (ng/mL)	50±2169	112±2549	17±1053	139±1719	13±284	338±4211
Hemoglobin (g/dL)	13.3±2.2	13.7±2.1	12.6±2.3	12.8±2.3	12.6±2.2	13.0±2.2
Platelet Count (k/μL)	138±78	119±63	177±101	128±69	164±92	127±65
Creatinine (mg/dL)	1.1±0.9	0.9±0.5	1.0±0.6	0.9±0.3	1.3±1.0	1.0±0.4
Bilirubin (g/dL)	1.5±1.9	1.4±1.5	2.4±3.5	2.0±2.5	1.2±1.3	1.2±0.9*
INR	1.3±0.4	1.2±0.3	1.4±0.5	1.3±0.3*	1.4±0.6	1.3±0.4
Albumin (g/dL)	3.3±0.7	3.3±0.6*	3.2±0.8	3.2±0.7*	3.5±0.7	3.5±0.6*
Alkaline Phosphatase (U/L)	118±70	123±59	147±103	132±67	124±96	121±73.3*
GGT (U/L)	205±299	209±236*	330±457	246±288	154±205	174±176*
AST/√ALT ratio	10.5±6.0	10.9±4.2	10.7±6.7	9.8±4.6	6.9±5.0	7.7±3.1

* All the difference between HCC and no-HCC patients were statistically significant at a p-value <0.05, EXCEPT the ones marked with an asterisk *

### Risk of HCC by cirrhosis etiology

**Fig 1:**
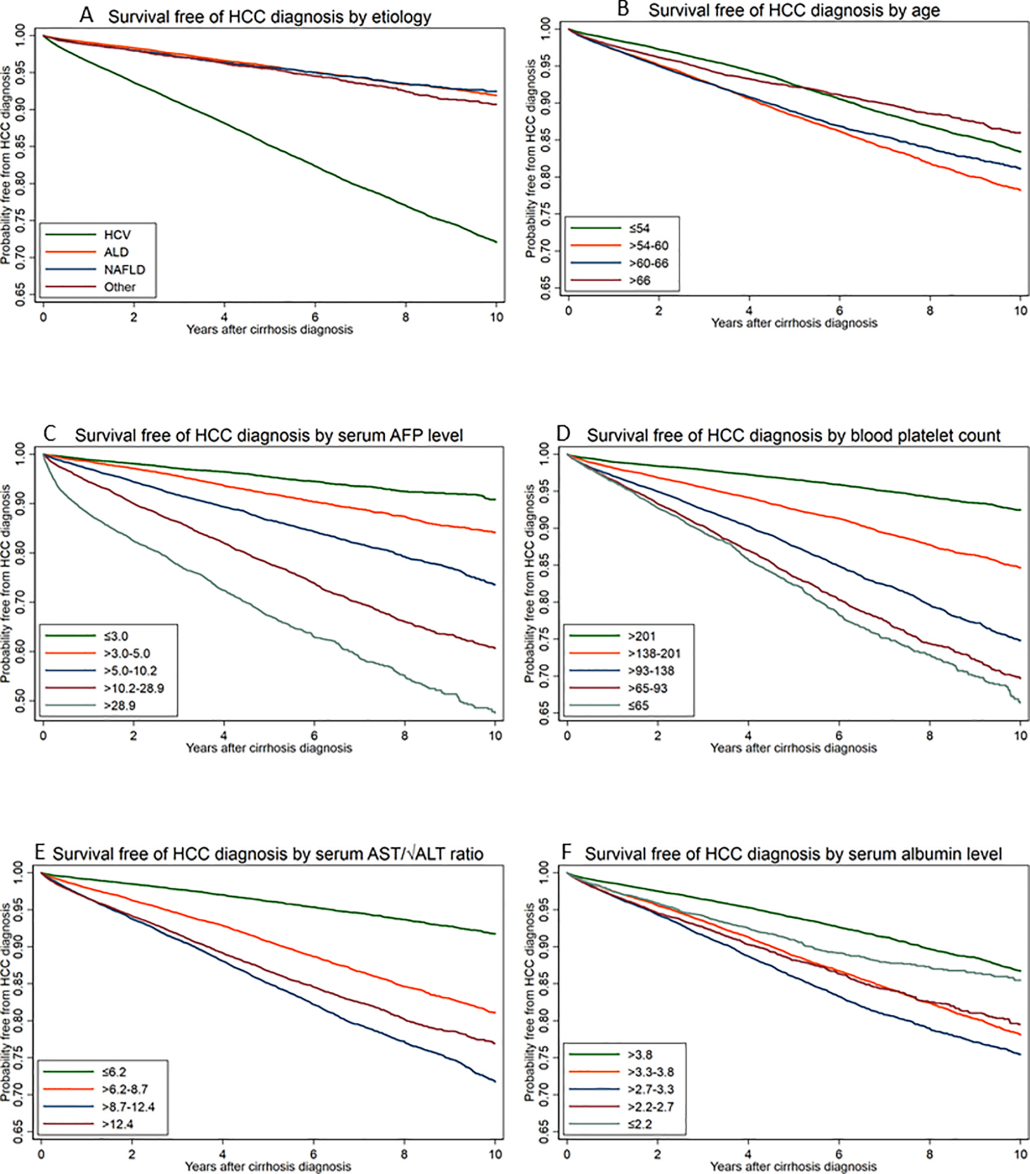
Kaplan-Meier curves showing the probability of being free of HCC after the diagnosis of cirrhosis, plotted by a. Etiology of cirrhosis. b. Age. c. Serum AFP level. d. Blood platelet count. e. Serum AST/√ALT ratio. f. Serum albumin level.

During a mean follow-up of 4.3 years, 10,042 new cases of HCC were diagnosed in 116,404 patients (incidence = 2.0 per 100 patient-years). Patients with HCV had more than 3 times higher incidence of HCC (3.3 per 100 patient-years) than patients with ALD (0.86 per 100 patient-years), NAFLD (0.90 per 100 patient-years) or OTHER (1.0 per 100 patient-years) etiologies of cirrhosis (**Table 3** and **Fig 1A**). Multivariable Cox proportional hazards analyses confirmed that compared to patients with HCV-cirrhosis, those with ALD-cirrhosis (adjusted hazard ratio [AHR] 0.30, 95% CI 0.28–0.32), NAFLD-cirrhosis (AHR 0.34, 95% CI 0.31–0.37), or OTHER-cirrhosis (AHR 0.40, 95% CI 0.36–0.44) had significantly lower risk of HCC after adjusting for baseline characteristics (**Table 3**). Competing risks analysis yielded very similar adjusted sub-hazard ratios (**Table 3**).

**Table 3:** Comparison of HCC risk after the diagnosis of cirrhosis, according to etiology of cirrhosis.

Etiology of Cirrhosis	Number of Patients	Patient- Years	Number with HCC	Incidence of HCC (per 100 patient-years)	Hazard Ratio	Adjusted Hazard Ratio*	Adjusted Sub-Hazard Ratio**
**A. 2001–2014**							
**ALL PATIENTS**	116,404	501,315	10,042	2.0	N/A	N/A	N/A	
**HCV**	52,671	229,448	7605	3.3	1	1	1	
**ALD**	35,730	160,639	1388	0.86	0.26 (0.25–0.28)	0.30(0.28–0.31)	0.33(0.3–0.36)	
**NAFLD**	17,354	67,881	608	0.90	0.27 (0.25–0.29)	0.33(0.30–0.36)	0.33(0.29–0.37)	
**OTHER**	10,649	43,346	441	1.0	0.31 (0.28–0.34)	0.39(0.36–0.44)	0.4(0.35–0.47)	
**HCV + ALD**	26,947	115,005	3850	3.3	1	1	1	
**HCV no ALD**	25,724	114,443	3755	3.3	0.98 (0.94–1.03)	1.02(0.98–1.07)	1.02(0.96–1.08)	
**B. 2001–2007†**							
**ALL PATIENTS**	55,138	182,724	2,841	1.55	N/A	N/A	N/A	
**HCV**	23,730	80,783	2120	2.62	1	1	1	
**ALD**	18,407	62,437	436	0.70	0.27(0.24–0.29)	0.31(0.28–0.35)	0.33(0.28–0.39)	
**NAFLD**	7,445	23,066	145	0.63	0.24(0.20–0.28)	0.31(0.25–0.37)	0.30(0.23–0.39)	
**OTHER**	5,556	16,439	140	0.85	0.32(0.27–0.38)	0.43(0.36–0.52)	0.42(0.32–0.56)	
**HCV + ALD**	11,891	40,351	1,016	2.52	1	1	1	
**HCV no ALD**	11,839	40,432	1,104	2.73	1.08(1.00–1.18)	1.16(1.06–1.28)	1.07(0.97–1.17)	
**C. 2008–2014†**							
**ALL PATIENTS**	61,266	184,203	4,553	2.47	N/A	N/A	N/A	
**HCV**	28,941	88,891	3482	3.92	1	1	1	
**ALD**	17,323	52,105	557	1.07	0.27(0.25–0.30)	0.31(0.28–0.34)	0.32(0.28–0.37)	
**NAFLD**	9,909	28,534	342	1.20	0.31(0.27–0.34)	0.39(0.34–0.44)	0.37(0.32–0.44)	
**OTHER**	5,093	14,673	172	1.17	0.30(0.26–0.35)	0.39(0.33–0.45)	0.37(0.3–0.46)	
**HCV + ALD**	15,056	45,628	1,856	4.07	1	1	1	
**HCV no ALD**	13,885	43,262	1,626	3.76	0.93(0.87–0.99)	0.98(0.91–1.05)	0.98(0.91–1.05)	

† Limited to a maximum follow-up of 5 years to make the two time periods (2001–2007 vs 2008–2014) more directly comparable.

* Adjusted by Cox proportional hazards analysis for etiology of cirrhosis, age, BMI, sex, race/ethnicity, decompensated cirrhosis, diabetes, platelet count, bilirubin, creatinine, albumin, hemoglobin and AST/√ALT ratio.

** Adjusted by competing risks proportional hazards analysis for the same characteristics listed above, with death and liver transplantation as competing risks to the diagnosis of HCC.

Among patients with HCV, there was no difference in HCC incidence or risk between those with and without a history of alcohol use disorders (**Table 3**), supporting our decision to consider patients with HCV infection as a single category irrespective of history of alcohol use.

When we divided patients into those diagnosed with cirrhosis in 2001–2007 and those diagnosed in 2008–2014, patients with cirrhosis due to ALD, NAFLD or Other etiologies had significantly lower HCC risk than those with HCV in both time periods (**Table 3**).

### Trends in HCC risk over time by cirrhosis etiology

**Fig 2:**
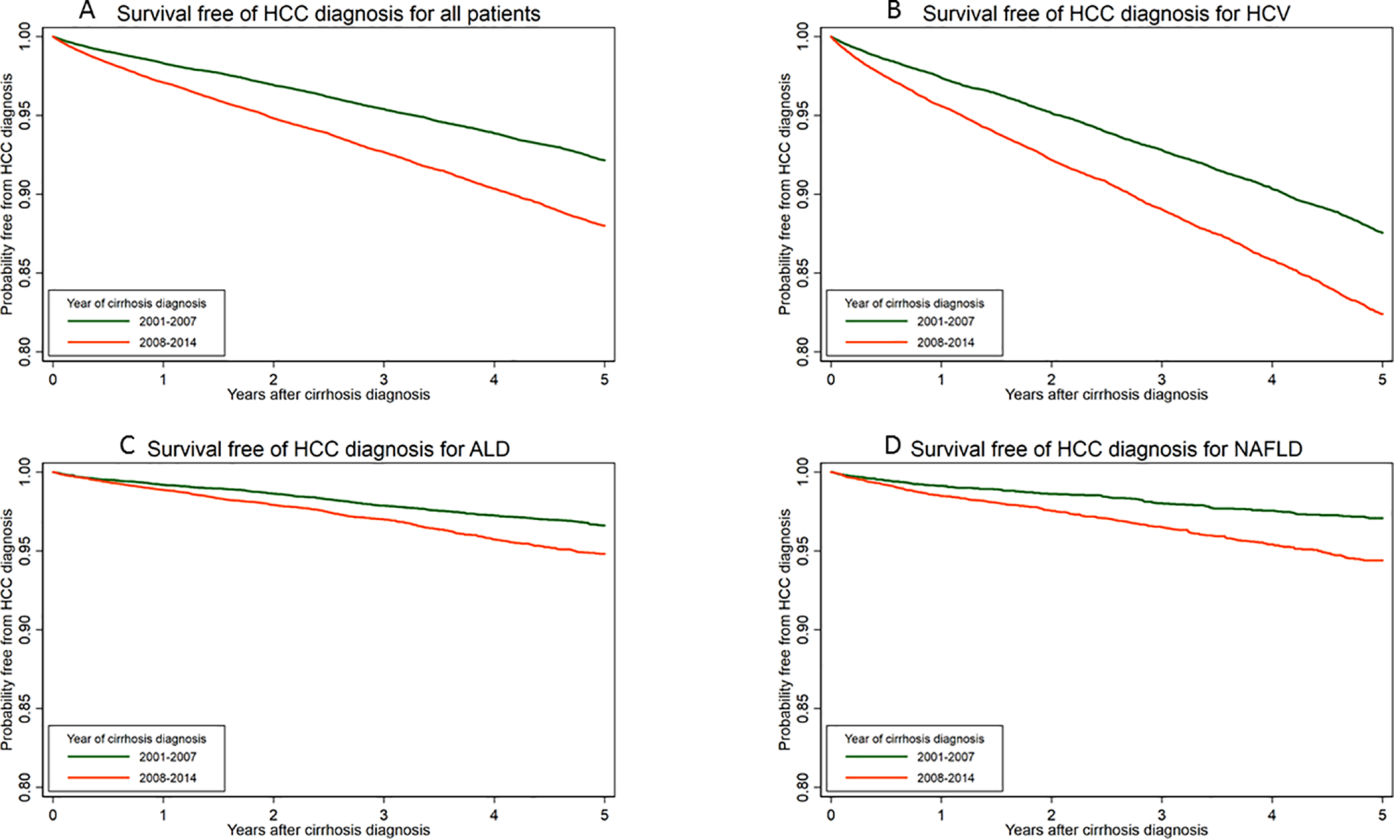
Kaplan-Meier curves showing the probability of being free of HCC after the diagnosis of cirrhosis, comparing patients diagnosed with cirrhosis in 2001–2007 to those diagnosed in 2008–2014. a. All patients. b. HCV. c. ALD. d. NAFLD.

HCC incidence was higher in patients with cirrhosis diagnosed in 2008–2014 (2.47 per 100 patient-years) compared to those diagnosed in 2001–2007 (1.55 per 100 patient-years) (**Table 4** and **Fig 2**). An increase in HCC incidence was observed among all etiologies of cirrhosis. After adjustment for baseline characteristics, the 2008–2014 cohort had higher HCC risk than the 2001–2007 cohort (AHR 1.38, 95% CI 1.32–1.45), an association that persisted among all cirrhosis etiologies. Competing risks analysis yielded very similar adjusted sub-hazard ratios (**Table 4**).

**Table 4:** Comparison of HCC risk among patients with cirrhosis diagnosed in 2008–2014 versus those diagnosed in 2001–2007†.

Year of Cirrhosis Diagnosis	Number of Patients	Patient- Years	Number with HCC	Incidence of HCC (per 100 patient-years)	Hazard Ratio	Adjusted Hazard Ratio*	Adjusted Sub-Hazard Ratio**
**ALL PATIENTS**							
**2001–2007**	55,138	182,724	2,841	1.55	1	1	1
**2008–2014**	61,266	184,203	4,553	2.47	1.59(1.52–1.67)	1.38(1.32–1.45)	1.36(1.27–1.46)
**HCV**							
**2001–2007**	23,730	80,783	2120	2.62	1	1	1
**2008–2014**	28,941	88,891	3,482	3.92	1.50(1.42–1.58)	1.31(1.23–1.39)	1.31(1.2–1.43)
**ALD**							
**2001–2007**	18,407	62,437	436	0.70	1	1	1
**2008–2014**	17,323	52,105	557	1.07	1.53(1.35–1.73)	1.23(1.08–1.41)	1.18(0.98–1.42)
**NAFLD**							
**2001–2007**	7,445	23,066	145	0.63	1	1	1
**2008–2014**	9,909	28,534	342	1.2	1.88(1.55–2.29)	1.44(1.18–1.77)	1.40(1.05–1.88)

† Limited to a maximum follow-up of 5 years to make the two time periods (2001–2007 vs. 2008–2014) more directly comparable

* Adjusted by Cox proportional hazards analysis for etiology of cirrhosis, age, BMI, sex, race/ethnicity, decompensated cirrhosis, diabetes, platelet count, bilirubin, creatinine, albumin, hemoglobin and AST/√ALT ratio.

** Adjusted by competing risks proportional hazards analysis for the same characteristics listed above, with death and liver transplantation as competing risks to the diagnosis of HCC.

The greatest increase over time occurred in patients with NAFLD-cirrhosis in relative terms (AHR 1.44, 95% CI 1.18–1.77) and patients with HCV-cirrhosis in absolute terms (from 2.62 to 3.92 per 100 patient-years).

### Risk factors for HCC by cirrhosis etiology (Table 5 and Table 6)

In univariable analyses (**Table 5**), the most important predictors of HCC among all liver disease etiologies were older age, male sex, Hispanic ethnicity, high serum AFP level, alkaline phosphatase level and AST/√ALT ratio, and low platelet count and serum albumin level (**Fig 1B–1F**).

**Table 5:** Univariable (unadjusted) associations between baseline characteristics and the risk of HCC in patients with cirrhosis, presented according to etiology of cirrhosis (HCV, ALD and NAFLD).

	ETIOLOGY OF CIRRHOSIS:		
	HCV N = 52,671	ALD N = 35,730	NAFLD N = 17,354
Patient Characteristics†	Crude Hazard Ratio	Crude Hazard Ratio	Crude Hazard Ratio
**Age**	1.21*	1.41*	1.27*
**BMI**	0.94*	1.35*	1.07
**Female vs. Male**	0.44*	0.11*	0.34**
**Race/Ethnicity (%)**			
White, non-Hispanic	1	1	1
Black, non-Hispanic	1.00	0.52*	0.34*
Hispanic	1.25*	2.11*	1.84*
Other	1.06	1.04	1.50
Declined to answer/missing	1.12**	1.01	1.06
**HCV Genotype (%)**			
Genotype 1	1	N/A	N/A
Genotype 2	0.72*	N/A	N/A
Genotype 3	1.44*	N/A	N/A
Genotype 4	0.75	N/A	N/A
**HIV co-infection**	0.68*	0.75	0.82
**HBV co-infection**	1.15	N/A	N/A
**Decompensated Cirrhosis**	1.21*	1.10	0.75**
**Ascites**	0.98	0.86	0.53*
**Encephalopathy**	1.05	1.09	0.58
**Gastroesophageal Varices with or without bleeding**	1.35*	1.38*	1.03
**Diabetes**	0.99	2.16*	2.23*
**Alcohol Use Disorder**	1.02	N/A	N/A
**Substance Use Disorder**	0.91*	0.52*	0.82
**Laboratory Results**			
Alpha Fetoprotein	1.65*	1.99*	2.10*
Hemoglobin	1.03*	1.03	1.02
Platelet Count	1.45*	1.80*	1.66*
Creatinine	0.92*	0.91*	0.77*
Bilirubin	1.23*	1.21*	1.30*
INR	1.16*	1.19*	0.95
Albumin	1.28*	1.15*	1.23*
Alkaline Phosphatase	1.34*	1.06**	1.22**
GGT	1.17*	0.97	1.33*
AST/√ALT ratio	1.43*	1.10*	1.58*

* p-value <0.001

** p-value 0.05–0.001

† For the continuous variables, we are presenting hazard ratios for each increasing quartile (for age, BMI, alpha fetoprotein, creatinine, bilirubin, INR, alkaline phosphatase, AST/√ALT ratio and GGT) and decreasing quartile (for hemoglobin, platelet count and albumin).

For the dichotomous variables, we are presenting the hazard ratios for the presence versus the absence of the characteristic (e.g. diabetes versus no diabetes).

**Table 6:** Independent risk factors for HCC in patients with cirrhosis derived from multivariable Cox proportional-hazards regression, presented according to etiology of cirrhosis (HCV, ALD and NAFLD).

	ETIOLOGY OF CIRRHOSIS:					
	HCV N = 44,007*		ALD N = 29,326*		NAFLD N = 13,456*	
Patient Characteristics‡	Adjusted† Hazard Ratio	P-value	Adjusted† Hazard Ratio	P-value	Adjusted† Hazard Ratio	P-value
**Age (yrs)**						
20–54	**1**		**1**		**1**	
>54–60	**1.55**	< 0.001	**1.8**	< 0.001	**2.29**	< 0.001
>60–66	**1.81**	< 0.001	**2.37**	< 0.001	**2.45**	< 0.001
>66	**1.85**	< 0.001	**2.81**	< 0.001	**2.71**	< 0.001
**Sex**						
Male	**1**		**1**		**1**	
Female	**0.5**	< 0.001	**0.35**	0.02	**0.4**	< 0.01
**Race/Ethnicity**						
White, non-Hispanic	**1**		**1**		**1**	
Black, non-Hispanic	**1.03**	0.37	**0.71**	0.01	**0.52**	0.03
Hispanic	**1.2**	< 0.001	**1.73**	< 0.001	**2.02**	< 0.001
Other	**1.13**	0.18	**1.11**	0.61	**1.82**	0.03
Declined to answer/missing	**1.1**	0.06	**0.97**	0.83	**1.02**	0.9
**BMI (Kg/m**^**2**^**)**						
≤18.0	**0.85**	0.38	**1.03**	0.93	**-**	-
>18.0–24.5	**1**		**1**		**1**	
>24.5–28.0	**1**	0.96	**1.59**	< 0.001	**1.2**	0.48
>28.0–32.0	**0.93**	0.04	**1.74**	< 0.001	**1.52**	0.08
>32.0	**0.84**	< 0.001	**1.88**	< 0.001	**1.45**	0.1
**HCV Genotype**						
1	**1**		**N/A**	N/A	**N/A**	N/A
2	**0.75**	< 0.001	**N/A**	N/A	**N/A**	N/A
3	**1.37**	< 0.001	**N/A**	N/A	**N/A**	N/A
4	**0.72**	0.17	**N/A**	N/A	**N/A**	N/A
**HIV co-infection**						
No	**1**		**1**		**1**	
Yes	**0.64**	< 0.001	**1.16**	0.8	**1.22**	0.74
**Diabetes**						
No	**1**		**1**		**1**	
Yes	**1.02**	0.6	**1.54**	< 0.001	**1.93**	< 0.001
**Alcohol Use Disorder**						
No	**1**		**N/A**	N/A	**N/A**	N/A
Yes	**0.97**	0.21	**N/A**	N/A	**N/A**	N/A
**Alpha Fetoprotein (ng/mL)**						
≤3.0	**1**		**1**		**1**	
>3.0–5.0	**1.9**	< 0.001	**1.46**	< 0.01	**1.05**	0.79
>5.0–10.2	**2.48**	< 0.001	**2.16**	< 0.001	**2.15**	< 0.001
>10.2–28.9	**3.51**	< 0.001	**4.47**	< 0.001	**6.49**	< 0.001
>28.9	**4.88**	< 0.001	**30.96**	< 0.001	**19.63**	< 0.001
**Platelet Count (k/μL)**						
>201	**1**		**1**		**1**	
>138–201	**1.47**	< 0.001	**2.09**	< 0.001	**1.89**	< 0.001
>93–138	**2.02**	< 0.001	**3.26**	< 0.001	**2.49**	< 0.001
>65–93	**2.32**	< 0.001	**4.48**	< 0.001	**3.68**	< 0.001
≤65	**2.38**	< 0.001	**4.79**	< 0.001	**3.75**	< 0.001
**Creatinine (mg/dL)**						
≤0.8	**1**		**1**		**1**	
>0.8–0.9	**0.98**	0.5	**0.94**	0.47	**1.23**	0.13
>0.9–1.16	**0.96**	0.18	**0.78**	< 0.01	**0.98**	0.88
>1.2–1.6	**0.94**	0.15	**0.71**	< 0.01	**0.54**	< 0.001
>1.6	**0.65**	< 0.001	**0.36**	< 0.001	**0.41**	< 0.001
**INR**						
≤1.1	**1**		**1**		**1**	
>1.1–1.2	**1.14**	< 0.001	**1.19**	0.12	**0.97**	0.85
>1.2–1.4	**1.03**	0.45	**1.32**	< 0.01	**0.78**	0.1
>1.4–1.8	**0.85**	< 0.01	**1.21**	0.1	**0.55**	0.01
>1.8	**0.77**	< 0.01	**0.91**	0.56	**0.48**	< 0.01
**Albumin (g/dL)**						
>3.8	**1**		**1**		**1**	
>3.3–3.8	**1.34**	< 0.001	**1.46**	< 0.001	**1.31**	0.02
>2.7–3.3	**1.55**	< 0.001	**1.65**	< 0.001	**1.37**	0.02
>2.2–2.7	**1.5**	< 0.001	**1.67**	< 0.001	**1.56**	0.02
≤2.2	**1.31**	< 0.001	**1.52**	< 0.01	**1.55**	0.11
**Hemoglobin (g/dL)**						
>15.8	**1**		**1**		**1**	
>14.5–15.8	**0.93**	0.09	**0.91**	0.48	**1**	0.98
>13.1–14.5	**0.85**	< 0.001	**0.82**	0.12	**0.82**	0.3
>11.1–13.1	**0.76**	< 0.001	**0.77**	0.03	**0.66**	0.03
≤11.1	**0.65**	< 0.001	**0.75**	0.03	**0.77**	0.19
**Alkaline Phosphatase (U/L)**						
≤81	**1**		**1**		**1**	
>81–111	**1.34**	< 0.001	**1.22**	0.04	**1.2**	0.15
>111–159	**1.59**	< 0.001	**1.39**	< 0.001	**1.43**	< 0.01
>159–235	**1.75**	< 0.001	**1.24**	0.05	**1.28**	0.15
>235	**1.49**	< 0.001	**0.92**	0.56	**1.27**	0.27
**AST/**√**ALT ratio**						
≤6.2	**1**		**1**		**1**	
>6.2–8.7	**1.63**	< 0.001	**1.87**	< 0.001	**1.78**	< 0.001
>8.7–12.4	**2.26**	< 0.001	**2.4**	< 0.001	**2.3**	< 0.001
>12.4	**2.36**	< 0.001	**1.31**	0.02	**2.61**	< 0.001
**GGT (U/L)**						
≤61	**1**		**1**		**1**	
>61–129	**1.42**	< 0.001	**1.18**	0.34	**1.35**	0.2
>129–286	**1.68**	< 0.001	**1.37**	0.05	**1.42**	0.17
>286–586	**1.75**	< 0.001	**1.14**	0.5	**2.01**	0.03
>586	**1.27**	0.05	**0.84**	0.46	**1.05**	0.93
**Bilirubin (g/dL)**						
≤0.7	**1**		**1**		**1**	
>0.7–1.1	**1.05**	0.19	**0.99**	0.92	**1.01**	0.96
>1.1–2.0	**1.04**	0.33	**1.13**	0.2	**1.19**	0.17
>2.0–3.9	**0.92**	0.1	**1**	0.99	**0.97**	0.85
>3.9	**0.77**	< 0.01	**0.73**	0.03	**0.66**	0.35

* The study population is lower than in Tables 1–3 because patients missing one or more of the laboratory tests that were simultaneously adjusted for were dropped from the multivariable analyses.

‡ All laboratory tests were categorized into 0-25^th^, 25^th^-50^th^, 50^th^-75^th^, 75^th^-90^th^and 90^th^-100^th^ percentiles. Age, AST/√ALT ratio and BMI were categorized into quartiles but for BMI an additional category of <18 Kg/m^2^ was included, as potentially abnormally low.

†Adjusted for age, sex, race/ethnicity, diabetes, BMI, albumin, platelet count and AST/√ALT ratio modeled as dummy-categorical variables.

**Table 6** shows only the characteristics that remained statistically significant after adjusting for the most important potential confounders (age, sex, race/ethnicity, diabetes, BMI, albumin, platelet count and AST/√ALT ratio) and categorizing continuous variables. The strongest predictors among all liver disease etiologies were: older age, male sex, Hispanic ethnicity, high serum AFP level, alkaline phosphatase level and AST/√ALT ratio, and low platelet count and serum albumin level.

Diabetes was significantly associated with HCC in patients with ALD and NAFLD-cirrhosis but not in patients with HCV-cirrhosis. Also, Hispanic ethnicity was much more strongly associated with HCC in patients with ALD and NAFLD-cirrhosis than in patients with HCV-cirrhosis. High BMI was associated with HCC in patients with ALD but not in patients with HCV or NAFLD. HCV genotype 3 infection was associated with significantly higher risk while genotype 2 infection was associated with lower risk, compared to genotype 1 infection. Certain ranges of high GGT were associated with HCC in patients with HCV (61–586 U/L) and NAFLD (286–586 U/L), but not in patients with ALD-cirrhosis.

Noteworthy but counterintuitive results included the association of HIV co-infection with *lower* HCC risk in patients with HCV-cirrhosis and the strong association of high serum creatinine with *lower* HCC risk among all cirrhosis etiologies.

In the analyses shown in **Table 6**, patients with missing data in one or more laboratory tests dropped out of the multivariable analyses–hence the sample size is slightly lower. We repeated the multivariable analyses including a missing category instead of dropping those with missing predictors and found almost identical results.

## Discussion

In a national cohort of cirrhotic patients, those with HCV-cirrhosis had approximately 3 times greater risk of HCC than those with NAFLD-cirrhosis or ALD-cirrhosis, after adjusting for baseline characteristics. HCC risk was 1.38 times higher in patients diagnosed with cirrhosis in 2008–2014 than those diagnosed in 2001–2007, an association that persisted among all cirrhosis etiologies. The most important risk factors for HCC irrespective of underlying cirrhosis etiology were older age, male sex, Hispanic ethnicity, high serum AFP level, alkaline phosphatase level and AST/√ALT ratio, and low platelet count and serum albumin level. Additionally, diabetes was a risk factor in patients with ALD and NAFLD and increased BMI in patients with ALD; neither diabetes nor increased BMI were risk factors for HCC in patients with HCV.

There was a remarkable increase in HCC incidence among all cirrhotic patients in the 7-year interval between the two cohorts that we compared (2001–2007 versus 2008–2014) from 1.55 to 2.47 cases per 100 patient-years. This increase was more pronounced in patients with HCV-cirrhosis and NAFLD-cirrhosis than in ALD-cirrhosis. Although this association was attenuated slightly by adjustment for baseline characteristics, it remained significant (AHR 1.38, 95% 1.32–1.45) suggesting that changes over time in baseline characteristics that we adjusted for–such as the “aging” of the HCV cohort[5], or the increasing prevalence of obesity and diabetes–do not entirely explain this trend. This suggests that unknown factors, other than the ones we adjusted for, might be causing this increase. However, it is impossible to exclude that increasing awareness of HCC over time or the introduction of the liver imaging reporting and data system (LI-RADS) in 2011 might have led to an increase in the rate of diagnosis without a true increase in incidence.

Our finding that HCV-related cirrhosis is independently associated with a 3-fold greater risk of HCC than ALD or NAFLD-related cirrhosis after adjusting for known risk factors suggests that the hepatitis C virus itself may have a direct carcinogenic effect[29–31]. HCV is an RNA virus without a DNA intermediate and, hence, does not integrate into the host genome. However, HCV viral proteins have been implicated in various oncogenic processes. In particular, the HCV core protein has been shown to play a key role in the downregulation of tumor suppressor genes, promoter activation of oncogenes, dysregulation of apoptosis, reactive oxidation species (ROS) formation, and immune modulation[32]. HCV may also play a role in inducing epigenetic changes associated with HCC. Specifically, hypermethylation of tumor suppressor genes and alteration of micro-RNA profiles implicated in hepatocarcinogenesis have been observed at higher frequencies in HCV-positive HCC than HCV-negative HCC[33]. In addition, the fact that genotype 3 HCV is associated with a greater risk of HCC than other genotypes suggests a direct carcinogenic effect of the virus.

Other mechanisms besides a direct viral carcinogenic effect could also explain the association between HCV-cirrhosis and HCC. Chronic inflammation and increased cell turnover are thought to drive HCC development in cirrhosis[30, 34]. There are important differences in the pathogenesis of necroinflammation between HCV, NAFLD and ALD, which may result in differences in HCC risk. Furthermore, in patients with HCV-related cirrhosis, the primary necro-inflammatory stimulus (i.e. the hepatitis C virus) is still present, whereas in ALD or NAFLD-related cirrhosis, the primary necro-inflammatory stimulus (i.e. alcohol use in ALD or a hypothesized lipotoxic molecule in NAFLD) may or may not still be present after the development of cirrhosis.

In patients who have HCV infection as well as a substantial history of alcohol use, it may be impossible to distinguish the etiology of cirrhosis, which is frequently labeled as “HCV plus alcohol”. However, our results clearly demonstrate that in cirrhotic patients with HCV infection, HCC risk is not influenced by a history of alcohol use disorders (**Table 3**). Stated differently, in cirrhotic patients with both HCV infection and alcohol use disorders, it is the HCV that determines HCC risk. Therefore, for the purposes of HCC risk estimation, cirrhotic patients with HCV can be categorized in a single group irrespective of prior alcohol use. This should be distinguished from ongoing alcohol use after the development of cirrhosis which may affect HCC risk but was not specifically studied in this report.

The most important risk factors for HCC among all etiologies of liver disease were older age, male gender, Hispanic ethnicity, high serum AFP, alkaline phosphatase, and AST/√ALT ratio, and low platelet count and serum albumin. Although some of these risk factors have been previously described [2, 24, 25, 27, 35, 36], others merit further discussion. Serum AFP has mostly been investigated as a *screening test for the detection* of HCC, the idea being that some HCCs secrete AFP. Serum AFP has only modest accuracy as a screening or diagnostic test for HCC and thresholds >20 ng/mL are most commonly recommended for screening[1, 37, 38]. However, we demonstrated that serum AFP level is a strong predictor of the *future development* of HCC and that even low levels of serum AFP, within the “normal range”, were strongly predictive. Compared to the baseline category of serum AFP ≤3.0 ng/mL (which was the lowest quartile), relatively low serum AFP levels in the second quartile (>3.0–5.0 ng/mL), the third quartile (>5.0–10.2), the 75^th^-90^th^ percentile (>10.2–28.9), as well as higher levels >90^th^ percentile (>28.9) were associated with high and progressively increasing HCC risk. It is very unlikely that these associations were caused by occult cancers that were present at baseline but only diagnosed later because we excluded all cancers diagnosed within the first 90 days and because the Kaplan-Meier curves of AFP categories continue to diverge from each other for many years of follow-up. Our results suggest that cirrhotic livers that produce higher baseline levels of AFP are at greater risk of developing HCC, for reasons that remain to be elucidated.

Low platelet count is one of the most consistently reported risk factors for HCC in cirrhotic patients[2, 39–41]. Low platelet count is a marker of more advanced cirrhosis. However, it is unlikely that this explains the association between thrombocytopenia and HCC because other markers of more advanced cirrhosis, such as hyperbilirubinemia or increased INR were not associated with HCC. Perhaps thrombocytopenia predicts HCC because it is a marker of portal hypertension and correlates with the hepatic venous portal gradient[42]. However, other signs of portal hypertension such as ascites and gastroesophageal varices were not significant predictors. Platelets, which cannot synthesize 5-hydroxytryptamine (5-HT), absorb 5-HT efficiently from the plasma pool and store it in their dense-body granules. It has been suggested that this platelet-derived 5-HT is important in liver regeneration[43]. It is tempting to speculate that a low platelet count results in a low level of platelet derived 5-HT which attenuates the ability of the liver to regenerate and thereby leads to increased risk of HCC.

AST/√ALT ratio, a component of the fibrosis-4 score, is considered to be a measure of fibrosis. Fibrosis development continues even after the development of cirrhosis and likely leads to worsening liver dysfunction. Therefore, even among cirrhotic patients, a high AST/√ALT ratio likely captures those with even more advanced fibrosis. The most plausible explanation for the strong association that we described between AST/√ALT ratio and HCC is that increasing fibrosis increases the risk of HCC. High AST/√ALT was associated with HCC risk in all etiologies of cirrhosis, though in ALD, its greatest risk peaked within the third quartile (8.7–12.4). This nonlinear association is likely explained by the fact that alcohol use acutely elevates AST. Thus, at very high levels (>12.4), the AST/√ALT ratio may reflect recent alcohol use and consequently become a less accurate predictor of fibrosis or HCC.

Some important differences between HCV, ALD and NAFLD in HCC risk factors may be particularly informative. Diabetes was associated with HCC risk in patients with ALD and NAFLD, but not in patients with HCV. High BMI was associated with HCC risk in patients with ALD, but not in patients with HCV (BMI is difficult to interpret in patients with NAFLD because, by definition, these patients had to have a BMI ≥30 kg/m^2^ or diabetes). Furthermore, Hispanic ethnicity was a much stronger risk factor for HCC in patients with ALD (AHR 1.73) or NAFLD (AHR 2.02) than in patients with HCV (AHR 1.2). Metabolic risk factors are common in Hispanics and may mediate the association between Hispanic ethnicity and HCC[44]. Collectively, these findings suggest that metabolic factors are much more important risk factors for HCC in NAFLD and ALD than in HCV-related cirrhosis.

Although HCV co-infection undoubtedly increases HCC risk in patients with HIV infection, whether HIV co-infection increases HCC risk in patients with HCV infection and cirrhosis is unclear. It has been hypothesized that HIV-related immune suppression might increase the risk of HCC[45]. However, a critical review of the literature did not reveal any clinical or epidemiological studies to support that HIV co-infection increases HCC risk in patients with HCV and cirrhosis after adjusting for potential confounders[46, 47]. We actually found the opposite association: HIV co-infection was associated with a lower risk of HCC in patients with HCV-cirrhosis. This could be due to unmeasured confounding such as differences in ongoing alcohol use or in the mode of transmission of HCV. Future studies need to confirm this controversial finding.

Serum creatinine concentration is a direct reflection of skeletal muscle mass, assuming constant glomerular filtration rate[48]. Sarcopenia is very common in patients with cirrhosis and strongly affects outcomes including survival in patients with cirrhosis and HCC[49]. It is plausible that the association that we found between high serum creatinine and low HCC risk reflects an association between low muscle mass and HCC risk. An association between sarcopenia and HCC risk has not previously been established and will require further confirmation.

### Limitations

Eradication of HCV reduces the risk of HCC[8]. For this reason, we censored patients with HCV-cirrhosis at the time they eradicated HCV. Therefore, our study reflects the incidence and risk factors of HCC in patients with HCV-cirrhosis who still have HCV infection. We are conducting separate studies specifically addressing the incidence and risk factors of HCC after eradication of HCV by antiviral treatment. The diagnoses of cirrhosis and HCC were based on ICD-9 codes recorded by the patients’ providers in their electronic medical records. These definitions of HCC and cirrhosis extracted from national VA datasets have been extensively validated and used in research. Prospective ascertainment of cirrhosis and HCC using *a priori* selected criteria is clearly not feasible in large, national studies with very long follow-up such as the one we conducted. Our results apply primarily to male patients with cirrhosis who constituted 97.5% of our study population–although the study population was so large that is still included a very large number of women (n = 2972) and the association between sex and HCC could be robustly ascertained. Substantial strengths of the study include the large sample size, large number of incident HCCs and long follow-up time. Data were available for most of the important potential risk factors for HCC. All patients were derived from a single, national healthcare system with fairly uniform practices and guidelines across its facilities.

In conclusion, our findings demonstrate the need for a more nuanced approach to HCC risk assessment among patients with cirrhosis. Current AASLD guidelines recommend a one-size-fits-all screening strategy for HCC (abdominal ultrasound with or without serum AFP every six months) in patients with cirrhosis[38], regardless of etiology or the presence or absence of various risk factors. However, HCC risk is very heterogeneous and critically depends on the predictors that we described. Our results also suggest that AFP can be a powerful predictor of HCC when used in combination with other risk factors, even if it is not adequate as a sole screening test. Investigators have recently attempted to develop algorithm-based risk models to estimate HCC risk and target high-risk patient populations for screening[26, 27, 35, 50]. Many of these models include AFP and other variables described in this study, and have shown early promise, though they have yet to be validated in clinical practice. Our study may help inform the future development of risk assessment models. Our results suggest that models that estimate HCC risk should be developed separately for patients with HCV, ALD and NAFLD-cirrhosis since they have different baseline risks as well as different predictors. Also, our results suggest that models developed using older data (e.g. data before 2008), will likely underestimate current HCC risk given the dramatic increase in incidence over time.

## Supporting information

S1 Table Definition of patient characteristics based on diagnostic ICD9 codes recorded at least twice in inpatient or outpatient records. (DOCX)

## Abbreviations

AHR: Adjusted hazard ratio
ALD: Alcoholic Liver Disease
ALT: Alanine aminotransferase
AST: Aspartate aminotransferase
BMI: Body mass index
HCC: Hepatocellular Carcinoma
HCV: Hepatitis C Virus
NAFLD: Nonalcoholic Fatty Liver Disease
VA: Veterans Affair
