# Supplementary material for: Differences in hepatocellular carcinoma risk, predictors and trends over time according to etiology of cirrhosis
Source: PLoS One. 2018 Sep 27;13(9):e0204412. doi: 10.1371/journal.pone.0204412 (PMC6160079; doi:10.1371/journal.pone.0204412)
Supplement: S1 Table — (DOCX) [file pone.0204412.s001.docx]

**Supplemental Table 1. Definition of patient characteristics based on diagnostic ICD9 codes recorded at least twice in inpatient or outpatient records**.

| **Characteristic** | **Definition** |
| --- | --- |
| Cirrhosis | 571.2 Alcoholic Cirrhosis Of Liver  571.5 Cirrhosis Of Liver Without Mention Of Alcohol  456.0 Esophageal Varices With Bleeding  456.1 Esophageal Varices Without Mention Of Bleeding  456.20 Esophageal Varices In Diseases Classified Elsewhere, With Bleeding  456.21 Esophageal Varices In Diseases Classified Elsewhere, Without Mention Of Bleeding  789.5 Ascites  567.23 Spontaneous Bacterial Peritonitis  572.2 Hepatic Coma or hepatic encephalopathy  070.44 Chronic hepatitis C with coma  572.4 Hepatorenal syndrome |
| Decompensated cirrhosis | 456.0 Esophageal Varices With Bleeding  456.1 Esophageal Varices Without Mention Of Bleeding  456.20 Esophageal Varices In Diseases Classified Elsewhere, With Bleeding  456.21 Esophageal Varices In Diseases Classified Elsewhere, Without Mention Of Bleeding  789.5 Ascites  567.23 Spontaneous Bacterial Peritonitis  572.2 Hepatic Coma or hepatic encephalopathy  070.44 Chronic hepatitis C with coma  572.4 Hepatorenal syndrome |
| Compensated cirrhosis | 571.2 Alcoholic Cirrhosis Of Liver  571.5 Cirrhosis Of Liver Without Mention Of Alcohol |
| Hepatocellular carcinoma | ICD9 155.0 Hepatocellular carcinoma  ICD10 C22.0 |
| Diabetes Mellitus Type 2 | 250.00-250.92 Diabetes |
| Alcohol Use Disorders | 305.0-305.03 alcohol abuse disorders  303.9-303.93 alcohol dependence  291.81 alcohol withdrawal  291.0 alcohol withdrawal delirium  291.8 other specified alcohol-induced mental disorders  291.9 unspecified alcohol-induced mental disorders  303.00 Acute alcohol intoxication  577 pancreatitis 2/2 etoh  357 alcoholic polyneuropathy  425.5 cardiomyopathy 2/2 alcohol  980.9 toxic effect of alcohol  305.00 Non-dependent alcohol abuse  571.0x alcoholic fatty liver  571.1x acute alcoholic hepatitis  571.3x alcoholic liver damage, unspec  571.2 alcoholic cirrhosis of liver |
| Hemochromatosis | 275.0 hemochromatosis (MUST exclude 275.1, 275.2 etc) |
| Primary Biliary Cirrhosis | 571.6 primary biliary cirrhosis |
| Autoimmune hepatitis | 571.32 autoimmune hepatitis |
| Primary sclerosing cholangitis | 576.1 cholangitis |

**Supplemental Table 2. Independent risk factors for HCC in patients with cirrhosis derived from multivariable Cox proportional-hazards regression, presented according to etiology of cirrhosis (HCV, ALD and NAFLD) – with categories added for missing values.**

|  | **ETIOLOGY OF CIRRHOSIS:** | | | | | |
| --- | --- | --- | --- | --- | --- | --- |
|  | **HCV**  **N=52,671** | | **ALD**  **N=35,729** | | **NAFLD**  **N=17,325** | |
| **Patient Characteristics*** | **Adjusted† Hazard Ratio** | **P-value** | **Adjusted† Hazard Ratio** | **P-value** | **Adjusted† Hazard Ratio** | **P-value** |
| **Age (yrs), quartiles** |  |  |  |  |  |  |
| 20-54 | 1 |  | 1 |  | 1 |  |
| >54 -60 | 1.54 | < 0.001 | 1.87 | < 0.001 | 2.51 | < 0.001 |
| >60-66 | 1.82 | < 0.001 | 2.41 | < 0.001 | 2.73 | < 0.001 |
| >66 | 1.77 | < 0.001 | 2.82 | < 0.001 | 2.86 | < 0.001 |
| **Sex** |  |  |  |  |  |  |
| Male | 1 |  | 1 |  | 1 |  |
| Female | 0.51 | < 0.001 | 0.34 | < 0.01 | 0.38 | < 0.01 |
| **Race/Ethnicity** |  |  |  |  |  |  |
| White, non-Hispanic | 1 |  | 1 |  | 1 |  |
| Black, non-Hispanic | 1.03 | 0.34 | 0.72 | < 0.01 | 0.53 | 0.02 |
| Hispanic | 1.18 | < 0.001 | 1.71 | < 0.001 | 1.99 | < 0.001 |
| Other | 1.11 | 0.23 | 1.02 | 0.9 | 1.75 | 0.02 |
| Declined to answer/missing | 1.09 | 0.07 | 1.04 | 0.69 | 1.04 | 0.76 |
| **BMI (Kg/m^2^)** |  |  |  |  |  |  |
| ≤18 | 0.76 | 0.12 | 0.98 | 0.96 | - | - |
| >18-24.5 | 1 |  | 1 |  | 1 |  |
| >24.5-28 | 1.01 | 0.83 | 1.59 | < 0.001 | 1.46 | 0.11 |
| >28 – 32 | 0.93 | 0.03 | 1.73 | < 0.001 | 1.73 | 0.01 |
| >32 | 0.84 | < 0.001 | 1.9 | < 0.001 | 1.67 | 0.02 |
| Missing | 0.21 | < 0.001 | 0.36 | 0.15 | 0.63 | 0.66 |
| **HCV Genotype** |  |  |  |  |  |  |
| 1 | 1 |  | N/A | N/A | N/A | N/A |
| 2 | 0.73 | < 0.001 | N/A | N/A | N/A | N/A |
| 3 | 1.41 | < 0.001 | N/A | N/A | N/A | N/A |
| 4 | 0.72 | 0.12 | N/A | N/A | N/A | N/A |
| Missing | 0.88 | < 0.001 | N/A | N/A | N/A | N/A |
| **HIV co-infection** |  |  |  |  |  |  |
| No | 1 |  | 1 |  | 1 |  |
| Yes | 0.64 | < 0.001 | 1 | 1 | 1.07 | 0.91 |
| **Diabetes** |  |  |  |  |  |  |
| No | 1 |  | 1 |  | 1 |  |
| Yes | 1.02 | 0.52 | 1.57 | < 0.001 | 1.95 | < 0.001 |
| **Alcohol Use Disorder** |  |  |  |  |  |  |
| No | 1 |  | N/A | N/A | N/A | N/A |
| Yes | 0.98 | 0.43 | N/A | N/A | N/A | N/A |
| **Alpha Fetoprotein (ng/mL)** |  |  |  |  |  |  |
| ≤3.0 | 1 |  | 1 |  | 1 |  |
| >3.0-5 | 1.73 | < 0.001 | 1.3 | 0.02 | 1.07 | 0.68 |
| >5.0-10.2 | 2.25 | < 0.001 | 1.97 | < 0.001 | 2.17 | < 0.001 |
| >10.2-28.9 | 3.08 | < 0.001 | 3.97 | < 0.001 | 7.3 | < 0.001 |
| >28.9 | 4.16 | < 0.001 | 32.2 | < 0.001 | 21.34 | < 0.001 |
| Missing | 1.76 | < 0.001 | 1 | 0.99 | 0.76 | 0.03 |
| **Platelet Count (k/µL)** |  |  |  |  |  |  |
| >201 | 1 |  | 1 |  | 1 |  |
| >138-201 | 1.49 | < 0.001 | 1.91 | < 0.001 | 1.98 | < 0.001 |
| >93-138 | 2.06 | < 0.001 | 3 | < 0.001 | 2.51 | < 0.001 |
| >65-93 | 2.38 | < 0.001 | 4.18 | < 0.001 | 3.64 | < 0.001 |
| ≤65 | 2.44 | < 0.001 | 4.57 | < 0.001 | 3.74 | < 0.001 |
| Missing | 1.82 | < 0.001 | 2.31 | < 0.001 | 2.23 | < 0.001 |
| **Creatinine (mg/dL)** |  |  |  |  |  |  |
| ≤0.8 | 1 |  | 1 |  | 1 |  |
| >0.8-0.9 | 0.98 | 0.57 | 0.95 | 0.54 | 1.12 | 0.38 |
| >0.9-1.16 | 0.96 | 0.17 | 0.81 | < 0.01 | 0.84 | 0.12 |
| >1.2-1.6 | 0.9 | 0.01 | 0.72 | < 0.001 | 0.51 | < 0.001 |
| >1.6 | 0.64 | < 0.001 | 0.45 | < 0.001 | 0.39 | < 0.001 |
| Missing | 0.94 | 0.3 | 0.7 | 0.01 | 0.79 | 0.25 |
| **INR** |  |  |  |  |  |  |
| ≤1.1 | 1 |  | 1 |  | 1 |  |
| >1.1-1.2 | 1.13 | < 0.001 | 1.27 | 0.02 | 1.02 | 0.87 |
| >1.2-1.4 | 1.04 | 0.3 | 1.34 | < 0.01 | 0.85 | 0.25 |
| >1.4-1.8 | 0.87 | < 0.01 | 1.2 | 0.09 | 0.56 | 0.01 |
| >1.8 | 0.78 | < 0.01 | 0.89 | 0.46 | 0.47 | < 0.01 |
| Missing | 0.89 | < 0.001 | 0.99 | 0.88 | 0.66 | < 0.001 |
| **Albumin (g/dL)** |  |  |  |  |  |  |
| >3.8 | 1 |  | 1 |  | 1 |  |
| >3.3-3.8 | 1.36 | < 0.001 | 1.52 | < 0.001 | 1.32 | 0.01 |
| >2.7-3.3 | 1.6 | < 0.001 | 1.7 | < 0.001 | 1.39 | < 0.01 |
| >2.2-2.7 | 1.53 | < 0.001 | 1.74 | < 0.001 | 1.53 | 0.02 |
| ≤2.2 | 1.34 | < 0.001 | 1.56 | < 0.001 | 1.61 | 0.07 |
| Missing | 1.2 | < 0.01 | 1.6 | < 0.001 | 0.99 | 0.94 |
| **Hemoglobin (g/dL)** |  |  |  |  |  |  |
| >15.8 | 1 |  | 1 |  | 1 |  |
| >14.5-15.8 | 0.92 | 0.05 | 0.99 | 0.97 | 1.04 | 0.83 |
| >13.1-14.5 | 0.85 | < 0.001 | 0.9 | 0.37 | 0.83 | 0.27 |
| >11.1-13.1 | 0.77 | < 0.001 | 0.84 | 0.14 | 0.68 | 0.03 |
| ≤11.1 | 0.68 | < 0.001 | 0.83 | 0.12 | 0.79 | 0.2 |
| Missing | 0.59 | < 0.001 | 0.9 | 0.64 | 0.25 | 0.03 |
| **Alkaline Phosphatase (U/L)** |  |  |  |  |  |  |
| ≤81 | 1 |  | 1 |  | 1 |  |
| >81-111 | 1.34 | < 0.001 | 1.22 | 0.02 | 1.31 | 0.02 |
| >111-159 | 1.59 | < 0.001 | 1.38 | < 0.001 | 1.52 | < 0.001 |
| >159-235 | 1.75 | < 0.001 | 1.28 | 0.01 | 1.33 | 0.08 |
| >235 | 1.51 | < 0.001 | 1.03 | 0.83 | 1.36 | 0.14 |
| Missing | 1.29 | < 0.001 | 1.02 | 0.88 | 1.28 | 0.22 |
| **AST/**√**ALT ratio, quantiles** |  |  |  |  |  |  |
| ≤6.2 | 1 |  | 1 |  | 1 |  |
| >6.2-8.7 | 1.63 | < 0.001 | 1.87 | < 0.001 | 1.85 | < 0.001 |
| >8.7-12.4 | 2.24 | < 0.001 | 2.35 | < 0.001 | 2.46 | < 0.001 |
| >12.4 | 2.34 | < 0.001 | 1.37 | < 0.01 | 2.69 | < 0.001 |
| Missing | 1.64 | < 0.001 | 1.3 | 0.05 | 1.49 | 0.02 |
| **GGT (U/L), quantiles** |  |  |  |  |  |  |
| ≤61 | 1 |  | 1 |  | 1 |  |
| >61-129 | 1.36 | < 0.001 | 1.24 | 0.18 | 1.28 | 0.25 |
| >129-286 | 1.56 | < 0.001 | 1.38 | 0.03 | 1.32 | 0.24 |
| >286-586 | 1.65 | < 0.001 | 1.19 | 0.32 | 1.95 | 0.01 |
| >586 | 1.1 | 0.42 | 0.77 | 0.2 | 1.13 | 0.8 |
| Missing | 1.33 | < 0.001 | 1.2 | 0.14 | 1.1 | 0.55 |
| **Bilirubin (g/dL), quantiles** |  |  |  |  |  |  |
| ≤0.7 | 1 |  | 1 |  | 1 |  |
| >0.7-1.1 | 1.07 | 0.04 | 1.02 | 0.83 | 1.05 | 0.67 |
| >1.1-2.0 | 1.06 | 0.11 | 1.2 | 0.04 | 1.22 | 0.09 |
| >2.0-3.9 | 0.96 | 0.34 | 1.07 | 0.49 | 1.1 | 0.57 |
| >3.9 | 0.8 | < 0.01 | 0.76 | 0.05 | 0.78 | 0.56 |
| Missing | 0.91 | 0.2 | 0.97 | 0.88 | 0.91 | 0.67 |

***All laboratory tests were categorized into 0-25^th^, 25^th^-50^th^, 50^th^-75^th^, 75^th^-90^th^and 90^th^-100^th^ percentiles. Age, AST/√ALT ratio and BMI were categorized into quartiles but for BMI an additional category of <18 Kg/m^2^ was included, as potentially abnormally low.**

**†Adjusted for age, sex, race/ethnicity, diabetes, BMI, albumin, platelet count and AST/√ALT ratio modeled as dummy-categorical variables.**
